# Determination of the social related factors of suicide in Iran: a systematic review and meta-analysis

**DOI:** 10.1186/1471-2458-13-4

**Published:** 2013-01-04

**Authors:** Milad Nazarzadeh, Zeinab Bidel, Erfan Ayubi, Khirollah Asadollahi, Kristin V Carson, Kourosh Sayehmiri

**Affiliations:** 1 Psychosocial Injuries Research Center, Ilam University of Medical Sciences, Ilam, Iran; 2 Student Research Committee, Ilam University of Medical Sciences, Ilam, Iran; 3 Department of Clinical Epidemiology, Faculty of Health, Ilam University of Medical Sciences, Ilam, Iran; 4 Department of Epidemiology, School of Public Health, Shahid Beheshti University of Medical Sciences, Tehran, Iran; 5 The Clinical Practice Unit, The Queen Elizabeth Hospital, Adelaide, Australia; 6 Department of social medicine, Faculty of Medicine, Ilam University of Medical Sciences, Ilam, Iran

**Keywords:** Meta-analysis, Social problems, Risk factors, Suicide attempt, Iran

## Abstract

**Background:**

Suicide, a social phenomenon, is a major health problem in most countries. Yet data relating to the role social factors play in the development of this condition are lacking, with some factors shrouded in greater ambiguity than others. As such, this review aimed to determine the prevalence of social-related factors resulting in suicide and to present these findings through meta-analyses, allowing for causes of heterogeneity to be examined.

**Methods:**

Scientific databases including PubMed and Science direct were searched using sensitive keywords. Two researchers reviewed the eligibility of studies and extracted data. Meta-regression with the Mantel-Haenszel method was conducted using a random effect model, in addition to subgroup analysis and Egger’s test.

**Results:**

A total of 2,526 articles were retrieved through the initial search strategy, producing 20 studies from 16 provinces for analysis. The most frequent cause of attempted suicide among the 20 analyzed articles was family conflict with 32% (95% CI: 26–38). Other related factors included marital problems (26%; 95% CI: 20–33), economic constrains (12%; 95% CI: 8–15) and educational failures (5%; 95% CI: 3–8). Results of meta-regression analysis found that sample size significantly affects heterogeneity for the factor ‘family conflict’.

**Conclusions:**

Social factors such as family conflicts and marital problems have a noticeable role in Iranian suicidology.

## Background

Suicide is an important cause of death around the world [1] and is considered to be a major health problem for most countries [2,3] imposing a substantial financial burden [4]. Current literature estimates the rate of attempted suicide in Iran to be 41.8 and 64.5 per 100,000 people for males and females respectively [5]. The World Health Organization reported that globally, approximately 815,000 people had committed suicide in the year 2000, with attempted suicide believed to be ten to twenty times higher for the same period [6].

The French sociologist, Émile Durkheim, was the first to consider that social determinants could be an integral factor of disease and to show that suicide is a social phenomenon [7,8]. Durkheim hypothesized that suicide had two important aspects, social integration and moral integration [9]. Feeling lonely, experiencing family conflicts and low levels of community integration are social factors revealed by western psychological theorists to be involved in the etiology of suicide [9]. However, these factors may vary significantly for different countries due to distinctions within social and cultural constructs.

Studies from developing countries reveal that stressful social events can be an important trigger for suicide attempts [10]. Moreover, there are reports that the family environment in particular [11] and quality of the marital relationship can be predictors of suicidal behavior [12,13]. The effect of pre-existing psychiatric disorders and past history of suicidal ideation are well recognized as predictors for future incidences [11]. However, there is a paucity of data examining the role of social risk factors in suicidal behavior for developing countries, resulting in a gap in the existing evidence.

Suicidal behavior is often the result of a complex matrix of clinical [14,15], familial [16], economic [17], political [18] and even geographical [19] variables. Indeed, recent studies are now beginning to observe a link between climate fluctuations and suicide [19-21]. However, none of these studies have established a definitive association between the climate and social related factors resulting in suicide, such as familial conflict. Iran in particular, is an arid to semi-arid region with significant climactic variations across each province [22]. With new evidence reporting even greater degrees of fluctuations in rainfall [23] and ground water levels [24,25] concerns are being raised over the psychological effect this may yield in the Iranian population.

This lack of precise and reliable evidence paring the social aspect of suicide with considerable climatic variations found in each Iranian province highlights a need for a comprehensive study to evaluate the available literature. Subsequently, the purpose of this study was to estimate the prevalence of social factors resulting in suicide in Iran through meta-analyses and to explore the causes of between-study variability.

## Methods

### Search strategy

All domestic scientific databases including Iranmedex, SID, Magiran, Irandoc, Medlib and IranPsych, as well as international databases including PubMed/Medline and ISI Web of Knowledge were searched for published data related to suicide in Iran. The search strategy was limited to the Persian and/or English language and articles published up until March 2012 were considered. Domestic scientific databases were searched only using the keyword ‘suicide’, as these databases do not distinguish synonyms from each other and do not allow sensitive search operation using linking terms such as ‘AND’, ‘OR’ or ‘NOT’. Consequently, this single keyword search was the most practical option. International databases were screened using the keywords ‘suicide’, ‘suicide attempt’ and ‘Iran’ using MeSH terms for standardization. The search string in PubMed was (("Suicide"[Mesh]) OR "Suicide, Attempted"[Mesh]) AND "Iran"[Mesh]).

### Selection and quality assessment of articles

Two researchers independently screened the titles of all retrieved citations, removing duplicate records and identifying potentially relevant studies for inclusion. Abstracts from selected citations were then independently reviewed by two researchers for further relevance, with full text manuscripts retrieved as appropriate. In the case of disagreement, a third assessor acted as a mediator. If the full text of an article could not be obtained, attempts were made to contact the study author. However, if this was unsuccessful the study was excluded from the analysis. EndNote X4 software was employed to screen citations from international databases.

Observational studies reporting the prevalence of social factors in suicide and/or attempted suicide in the form of published data, unpublished ‘raw’ data or as written reports were included in this review. The following study exclusion criteria were also applied: 1) inappropriate design i.e., studies with a suicide theme that did not examine social factors, or studies examining experimental designs or methodological quality or homicide studies, 2) inadequate reporting of results i.e., studies not reporting prevalence data for relevant outcomes, and 3) poor quality i.e., STROBE checklist score’s below 7.75 [26].

### Data extraction, management and definitions

The following data was extracted from included studies: first author, year of study, sample size, type of data collection, results of attempt (fatal, nonfatal, both), duration of study, type of climate, sex ratio, STROBE score and the prevalence of social risk factors related to suicide. The primary outcome measures of interest for this review are the prevalence of important social risk factors related to suicide and their 95% confidence interval. These social risk factors include: 1) family conflict, defined as a conflict within a family between husband and wife, parents and children, between siblings, between children or with extended families (grandparents, aunts, uncles, etc.), 2) marital problems, defined as any conflict between spouses (only among legally married couples i.e., not ‘defacto’ or ‘concubine’ relationships), 3) educational failure, which include all levels of educational failure for this definition, and 4) economic constrains, defined as: a) situational, that is it will vary for different people with different jobs/business e.g., customers stop spending money on luxuries items due to a recession resulting in a business loosing sales and subsequently profits, b) bankruptcy, that is the state of being unable to pay debts, and c) poverty, that is people producing an income below the required level to meet their basic needs and demands (this category does not just include people with a lack of food and housing).

### Methods of analysis

Data synthesis occurred through meta-analyses using the random effect model of Mantel-Haenszel, with available data presented in a Forest plot. Variance for each study was calculated using the binomial distribution formula. The presence of heterogeneity was determined by the chi^2^ test with a significance level of <0.1 combined with an I^2^ statistic for estimates of inconsistency within the meta-analyses. The I^2^ statistic estimates the percent of observed between-study variability due to heterogeneity rather than to chance and ranges from 0 to 100 percent. A value of 0% indicates no observed heterogeneity whilst 100% indicates significant heterogeneity. For this review we determined that I^2^ values above 75 percent were indicative of significant heterogeneity warranting analysis with a random effect model as opposed to the fixed effect model to adjust for the observed variability. This heterogeneity was further explored through subgroup analyses and meta-regression. A univariate and multivariate approach were employed to assess the causes of heterogeneity among the selected studies and the Egger test was conducted to examine potential publication bias. All analyses were conducted using Stata version 11.2 (Stata Corp LP, College Station, Texas) with ‘metan’, ‘metareg’ and ‘metabias’ comments.

## Results

**Figure 1:**
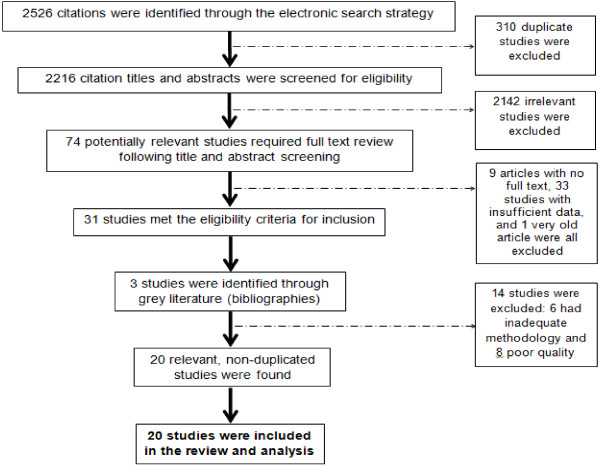
Quorum flow chart of the reviewing process for suicide attempts related to social factors in Iran.

A total of 2,526 citations were retrieved through electronic database screening, producing a total of 20 studies from 16 provinces of Iran that met all of the eligibility criteria [27-46]. The characteristics of each included study are reported in Table 1 and a Quorum flow chart outlining the details related to the selection process are presented in Figure 1. A total of 12,005 subjects were available for assessment from the 20 included studies. Overall the STROBE methodological quality assessment produced a maximum score of 31 and mean of 13.26.

**Table 1:** Characteristics of included studies

**Province**	**First author (year)**	**Sample size**	**Duration of study (month)**	**Suicide result**^**a**^	**Data collection procedure**	**Quality**^**b**^	**Men (%)**
Kohgiluyeh and Boyer-Ahmad	Abasi (1992)	154	12	D - N	Hospital	Low	18
Ilam	Keikhavani (1997)	266	12	D - N	Forensic medicine	Medium	36
Lorestan	Koldi (1999)	103	30	D	Unknown	Low	33
West Azerbaijan	Salary (2001)	4015	12	D -N	Hospital- Forensic medicine	Medium	36
Kerman	Zohor (2001)	44	1.5	N	Unknown	Medium	36
Mazandaran	Esmailnia (2001)	136	Unknown	D - N	Hospital	Medium	0
	Zarghami (2008)	100	Unknown	N	Hospital	High	Unknown
East Azerbaijan	Khazae (2001)	301	6	N	Hospital	Medium	44
Semnan	Zafarghandi (2002)	383	12	N	Hospital	Low	Unknown
South Khorasan	Mehran (2002)	50	9	N	Unknown	Medium	30
Gilan	Rahbar (2002)	240	4	N	Hospital	Low	41
Razavi Khorasan	Mohamadi (2003)	207	6	N	Hospital	Low	31
	Kosha (2008)	106	Unknown	N	Hospital	Medium	49
Ardabil	Molavi (2003)	218	6	N	Hospital	Medium	39
	Ghamari (2008)	42	24	N	Court records	Medium	81
Tehran	Karami (2003)	83	24	N	Hospital	Medium	3
Alborz	Nojomi (2003)	632	12	N	Hospital	Medium	38
National	Shirzad (2004)	260	6	D	Forensic medicine	Medium	69
Qazvin	Eslami (2005)	575	24	N	Hospital	Medium	30
Markazi	Rafiae (2007)	4226	24	D - N	Public health service	High	40

a. D: fatal suicide, N: nonfatal suicide, D - N : both fatal and nonfatal suicide.

b. Score of STROBE checklist. Under 7.75 = excluded; among 7.75 to 15.5: low; among 15.5 to 23.5: medium; higher than 23.5: High.

**Figure 2:**
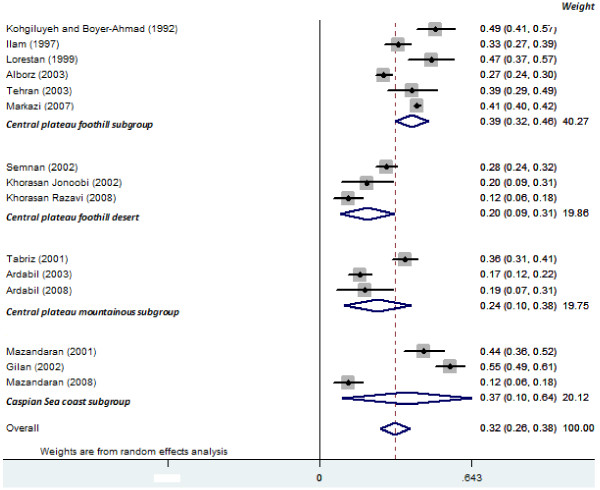
**Forest plot of studies related to family conflicts prevalence in attempted suicides in Iran.** Data are presented separately for Iran’s climates. Rectangles indicate point prevalence and size of the rectangles represent the weight given to each study in analysis; diamonds and the vertical dashed line indicate the combined point prevalence and horizontal lines indicate 95% confidence intervals.

### Family conflicts

**Figure 3:**
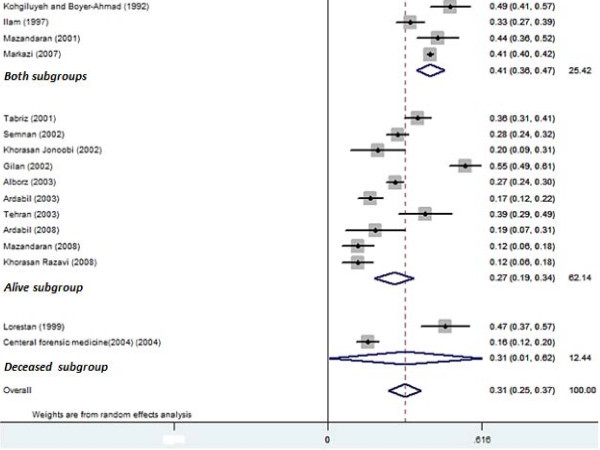
**Forest plot of family conflicts prevalence in attempted suicides in Iran.** Data are presented separately for results of attempts (alive, deceased, and both).

Among the 20 included studies 16 reported information related to family conflicts and were included in the assessment. Overall, prevalence estimates of family conflicts were 32% (95% CI: 26 to 38) (presented in Figure 2). The highest prevalence was reported from the Gilan province in 2002 with 55% (95% CI: 49 to 61) and the lowest prevalence reported from Razavi Khorasan in 2008 with 12% (95% CI: 6 to 18). Significant heterogeneity was observed between studies (Q = 386, p = 0.001, I^2^ = 96.4%) and consequently the random effect model was employed for the meta-analysis. Causes of the observed variability were assessed through meta-regression producing a significant effect for sample size (β = 0.00002, p = 0.02; Table 2) and a trend toward significance for climate (β = −0.02, p = 0.06; Figure 2). The prevalence of family conflicts in the central plateau foothills was higher than for any other climate (39%, 95% CI: 32 to 46). Moreover, subgroup analysis based on the results of suicide (fatal, nonfatal, both) produce evidence of significant difference between these subgroups (Figure 3). A potential publication bias was detected for family conflicts (Egger’s test β_0_: 0.40; p < 0.001).

**Table 2:** **Meta regression**^**a **^**of suspected variables of heterogeneity in univariate and multivariate models**

**Variables**	**Univariate**		**Multivariate**	
	**β**	**P-value**	**β**	**P-value**
Sample size	0.00002	0.44	0.0001	0.02
Climate ^b^	−0.02	0.45	−0.05	0.06
Year of study	−0.01	0.04	−0.01	0.14
Men ratio	−0.19	0.04	−0.10	0.34
Study population ^c^	0.07	0.19	−0.05	0.35
STROBE score	−0.01	0.17	−0.01	0.12

a. Moment base method.

b. Variables code includes: 1. Caspian sea coast; 2. Central plateau mountains; 3. Central plateau foothills; 4. Central plateau desert.

c. Alive people (non-fatal suicide), deceased people (fatal suicide), and both.

### Marital problems

Nine studies were available to assess marital problems with the highest prevalence reported from the Semnan province in 2002 with 38% (95% CI: 33 to 43) and the lowest prevalence reported from Razavi Khorasan in 2008 with 15% (95% CI: 8 to 22). Overall, the prevalence of marital problems was 26% (95% CI: 20 to 33) and the studies showed high levels of heterogeneity when pooled (Q = 84.83, p = 0.001, I^2^ = 90.6%). No between group differences were observed for climate or the result of attempted suicide through subgroup analyses or meta-regression. A significant publication bias was detected (Egger’s test β_0_: 0.32; p = 0.005).

### Educational failures

Meta-analysis included 12 studies and produced a prevalence of 5% (95% CI: 3 to 8) with the highest value reported from Razavi Khorasan province with 18% (95% CI: 7 to 29) and the lowest by Alborz province with 15% (95% CI: 12 to 18), although significant heterogeneity was observed (Q = 227.58, p = 0.001, I^2^ = 95.6%). No statistically significant differences were observed for subgroup analyses based on climate and outcome of attempted suicide or meta-regression for any variables. There was no evidence of publication bias (Egger’s test β_0_: 0.009; p = 0.17).

### Economic constraints

Meta-analysis for economic constraints was able to be assessed in 12 of the 20 included studies produced an average prevalence of 12% (95% CI: 8 to 15) with the highest prevalence reported from the South Khorasan province in 2002 with 40%, (95% CI: 26 to 54) and the lowest reported from Ilam in 1997 with 4% (95% CI: 2 to 6). Significant heterogeneity was observed in the meta-analysis (Q = 105.11, p = 0.001, I^2^ = 82.5%). No statistically significant differences were observed for subgroup analyses based on climate and outcome of attempted suicide or meta-regression for any variables. Significant publication bias was observed (Egger’s test β_0_: 0.04; p = 0.001).

## Discussion

This review of 20 studies found that social factors contributing to attempted suicide include family conflicts (30%), marital problems (26%), economic constrains (12%) and educational failures (5%), with family conflicts found to be the most prevalent for this Iranian study population. Results of the meta-regression also found that climate types and sample size produced significant levels of heterogeneity (Table 2). Subgroup analysis based on the type of climate showed an interaction with family conflict and the central plateau foothills area, more so than with any other climate in the country (Figure 2). A possible explanation might be that with the exception of Tehran (Capital of Iran), provinces in this subgroup are considered disadvantaged, particularly over the past twenty years. In addition, three studies were conducted over twelve years ago (between 1992 and 1999). With the rapid growth of public health in Iran in recent years, it seems likely that this higher prevalence of family conflicts from the central plateau foothills can potentially be attributed to the accumulation of older studies in this subgroup rather than climate type. Following a sensitivity analysis and the removal of the Mazandaran 2008 study (due to a small sample size; n = 100), subgroup analysis revealed a significant difference between family conflicts and climate for the region of the Capsian sea coast. Capsian sea coast located to the north of Iran have a largely temperate climate, with the Alborz mountain range in the surrounding area causing the terrain to be dominated by cloud-cover and consistent rainfall. Subsequently, our findings support the growing evidence of a relationship between human mood and the seasons [47-49]. A biological explanation may relate to the photoperiodic mechanisms of the sun’s rays [50], with new evidence indicating that serotonin transporter availability is altered in response to diminished light exposure with lower levels of serotonin found in winter months [51]. The amount of daily sunshine and global radiation can influence serotonin-1A receptor binding in the limbic brain regions of health subjects, highlighting a relationship between seasonal factors and the regulation of serotonergic transmissions [51]. Further research is required to determine the degree to which this potential relationship between exposure to sunlight and suicidal behavior exists. Using the results of suicide attempts, subgroup analysis suggests that family conflicts were less prevalent among people who survived from the suicide attempt (nonfatal), indicating that family may have an important role in the outcome. However, only two studies included fatal suicide with the meta-analysis producing high levels of heterogeneity. Consequently, a comparison between these subgroup was difficult highlight a paucity of data and as such the results should be interpreted with caution.

The meta-analysis examining the prevalence of marital problems in cases of attempted suicide produced findings similar to those reported by Janghorbani et al., who found that completed suicide and attempted suicide were more frequent among married couples than single people [5]. This is in contrast to the popular belief that marriage has a protective effect for against psychological or behavioral disorders [5]. Studies are required to investigate the particular effects of marriage itself, or other variables such as culture, economy and psychological factors on suicide attempts. The notable finding in this study was that all married subjects in these nine studies had nonfatal suicide attempts. A probable justification for this might be that married couples use less dangerous methods in attempting suicide, as their primary aim is to invoke a response from their spouse rather than actually complete suicide. These results may be influenced by the predominantly female representation in our study, which is however consistent with more women attempting suicide than men [5,52,53].

Unexpectedly, 5% of suicide attempts were found to be because of educational failures (95% CI: 3 to 8%). Previous studies report that people who attempt suicide following educational disappointments are usually aged between 14 and 17 years [54]. Student deaths impose a considerable burden on the society, and as such special attention to this issue is warranted. Due to inadequate reporting of results we were unable to distinguish high school students from university students, thus the 5% prevalence could be attributed to failure in high school exams or university entrance exams. This has the potential to be an important risk factor for many college entrance students taking exams (Konkor) as parents, communities and the individuals themselves place a significant amount of pressure on success for Iranian students in particular. Mental disorders such as depression should be considered as another risk factor for suicide attempts among Iranian students [55-59]. Nowadays, mental disorders are considered to be one of the most important causative factors related to suicide attempts [60-63]. There is a possibility that an interaction between mental disorders and educational failures may have a considerable impact on suicide attempts and further research such as case–control studies are needed to address this interaction. In addition, screening for mental disorder among students should be considered as part of standard care in schools and universities to allow early intervention to occur. Educational failures were highest in the Razavi Khorasan province with 18% and Alborz province with 15%, as such future research should target schools in these areas.

The relationship between socio-economic status and suicide has been confirmed through the existing literature [64] as has the subsequent impact on health. The results of this meta-analysis indicate a high prevalence of suicide due to economic constrains with a prevalence of 12% found across the twelve studies. Recent evidence from Iran has found that suicide attempts among young people more frequent than among elders with a negative relationship observed between suicide attempts and age [5]. The most important economic constraint among young people in Iran is likely to be unemployment. Therefore, job creation and encouragement to enter into the workforce particularly for among young people may result in a decrease in attempted suicides.

This study does have several limitations with all pooled analyses containing significant heterogeneity and subsequently should be interpreted with caution. The results should however be considered generalisable as they include a broad geographical cross-section from Iran. Potential factors contributing to the variability include location (setting), time of the study and characteristics of the population. Such heterogeneity is to be expected though considering the diverse cultures and ethnic groups found in Iran. Although many would argue that in the presence of such significant heterogeneity a meta-analysis should not be presented, we believe that providing the reader with the pooled prevalence estimates and a caution relating to the presence of heterogeneity will allow them to obtain a broad perspective examining the social factors related to suicide. Another limitation relates to the possibility that data evaluating risk factors for attempted suicide could be missed due to inadequate reporting of results or a lack of publication. Indeed, publication bias was detected for the majority of outcomes assessed in this study. The search strategy was also limited due to the use of standard search keywords in domestic databases and for this reason all synonyms were retrieved in both Persian and English languages. Moreover, a lack of comprehensive coverage for university databases containing research projects and theses further limit the search strategy. However, we believe that this review still provides the reader with an overview of the current available evidence and highlights that there is a potential gap due to reporting biases that need to be considered in future investigations and research.

Suicide is a multi-factorial problem with socio-related factors interacting reciprocally amongst each other with a great deal of overlap between these variables. Therefore, quantification of the association between these factors is now needed and should be the focus of future studies using case–control designs and meta-analyses reporting odds ratio with pooled interactions.

## Conclusion

In conclusion, the results of this meta-analysis indicate that family conflicts are a significant factor associated with suicide in Iran. The area most affected were provinces bordering the Caspian sea coast with subgroup analysis suggesting that climate may be an integral factor contributing to this observation. Returning to the question posed at the beginning of this study, this review has found that social factors have a perceptible role in Iranian suicidology.

## Abbreviations

SID: Scientific Information Database; Medlib: Medical library; STROBE: Strengthening the Reporting of Observational Studies in Epidemiology

## Competing interests

The authors declare that they have no competing interests.

## Authors’ contributions

All authors participated equally in the design of the study and in the interpretation of the data and the exchange of ideas during the study. MN and KS performed the statistical analysis. ZB and EA conducted literature searches and provided summaries of previous research studies and study selection. All authors participated equally in the manuscript writing. KA and KC checked and revised the grammatical and syntax errors. All authors read and approved the final manuscript.

## Pre-publication history

The pre-publication history for this paper can be accessed here:

http://www.biomedcentral.com/1471-2458/13/4/prepub
